# Comment on “Exotropia Is the Main Pattern of Childhood Strabismus Surgery in the South of China: A Six-Year Clinical Review”

**DOI:** 10.1155/2016/8251629

**Published:** 2016-07-25

**Authors:** Onder Ayyildiz, Osman Melih Ceylan, Fatih Mehmet Mutlu

**Affiliations:** ^1^Department of Ophthalmology, GATA Medical School, Ankara, Turkey; ^2^Department of Ophthalmology, Inonu University, Malatya, Turkey

We congratulate Yu et al. for their successful results where they evaluated the pattern distribution and the changes of strabismus surgery in pediatric population [1]. The authors observed that surgery for childhood exotropia was more common than surgery for esotropia in China and they stated that strabismus type and onset age were found to be associated with binocular function for subjects with older onset age and intermittent exotropia.

Intermittent exotropia (X(T)) is the most common form of childhood exotropia [2]. It has various nonsurgical treatment options such as patching, orthoptic therapy, and over-minus lenses; however, surgical treatment is the major curative option [3]. The type of X(T) and the timing of surgery for pediatric patients are important because of the risk of developing a suppression scotoma and monofixation esotropia, which can lead to loss of stereopsis and amblyopia [4]. In the present study, the authors did not mention strabismus subtypes; it would be better to establish the strabismus subtypes per age groups. We also kindly ask what inclusion and exclusion criteria were followed to decide surgery for both exotropia and esotropia. We noticed that the authors evaluated only near stereopsis with Titmus test. It was believed that distance stereoacuity and control of strabismus are more objective to decide the timing of surgery [5]. We kindly suggest that the authors evaluate the changes in binocular visual function in patients with X(T) of different ages preoperatively and postoperatively.
